# A Recombinant Vaccine-like Strain of Lumpy Skin Disease Virus Causes Low-Level Infection of Cattle through Virus-Inoculated Feed

**DOI:** 10.3390/pathogens11080920

**Published:** 2022-08-16

**Authors:** Irina Shumilova, Alexander Nesterov, Olga Byadovskaya, Pavel Prutnikov, David B. Wallace, Maria Mokeeva, Valeriy Pronin, Aleksandr Kononov, Ilya Chvala, Alexander Sprygin

**Affiliations:** 1 Federal State-Financed Institution, Federal Center for Animal Health, 600901 Vladimir, Russia; 2 Agricultural Research Council–Onderstepoort Veterinary Institute, Private Bag X5, Onderstepoort, Pretoria 0002, South Africa; 3 Department Veterinary Tropical Diseases, Faculty of Veterinary Science, University of Pretoria, Private Bag X4, Onderstepoort, Pretoria 0002, South Africa

**Keywords:** lumpy skin disease, virus, recombinant, transmission

## Abstract

Since 1989, lumpy skin disease of cattle (LSD) has spread out of Africa via the Middle East northwards and eastwards into Russia, the Far East and South-East Asia. It is now threatening to become a worldwide pandemic, with Australia possibly next in its path. One of the research gaps on the disease concerns its main mode of transmission, most likely via flying insect vectors such as biting flies or mosquitoes. Direct or indirect contact transmission is possible, but appears to be an inefficient route, although there is evidence to support the direct contact route for the newly detected recombinant strains first isolated in Russia. In this study, we used experimental bulls and fed them via virus-inoculated feed to evaluate the indirect contact route. To provide deeper insights, we ran two parallel experiments using the same design to discover differences that involved classical field strain Dagestan/2015 LSDV and recombinant vaccine-like Saratov/2017. Following the attempted indirect contact transmission of the virus from the inoculated feed via the alimentary canal, all bulls in the Dagestan/2015 group remained healthy and did not seroconvert by the end of the experiment, whereas for those in the Saratov/2017 recombinant virus group, of the five bulls fed on virus-inoculated feed, three remained clinically healthy, while two displayed evidence of a mild infection. These results provide support for recombinant virus transmission via the alimentary canal. In addition, of particular note, the negative control in-contact bull in this group exhibited a biphasic fever at days 10 and 20, developed lesions from day 13 onwards, and seroconverted by day 31. Two explanations are feasible here: one is the in-contact animal was somehow able to feed on some of the virus-inoculated bread left over from adjacent animals, but in the case here of the individual troughs being used, that was not likely; the other is the virus was transmitted from the virus-fed animals via an airborne route. Across the infected animals, the virus was detectable in blood from days 18 to 29 and in nasal discharge from days 20 to 42. Post-mortem and histological examinations were also indicative of LSDV infection, supporting further evidence for rapid, in F transmission of this virus. This is the first report of recombinant LSDV strain transmitting via the alimentary mode.

## 1. Introduction

Lumpy skin disease (LSD) is an OIE-reportable viral disease inflecting considerable losses on farms and affecting international trade [1]. The etiological agent is a capripoxvirus from the genus *Capripoxvirinae*, family *Poxviridae* [2]. The genome is a double-stranded DNA of about 156 kb [2,3]. LSD virus (LSDV) naturally infects cattle and water buffaloes, often leading to high morbidity and low mortality [4,5,6,7]. The typical clinical symptoms are skin lumps (nodules/lesions) that develop 5–7 days post-infection, turning into crusts, followed by necrosis and sloughing of the crusts (skin sequesters or sitfasts) leaving open lesions, which can serve as routes for secondary bacterial infections. The infected animals may also exhibit fever, excessive nasal and conjunctival discharge, oedema, abortion and a temporary drop in milk production [8]. Excreted fluids and skin lesions shed the virus in high quantities, which allows for environmental contamination and proves to be attractive food for flies [9]. The disease has two forms of clinical and subclinical manifestation [10].

Lumpy skin disease has recently received much interest after it began a rapid and aggressive spread northwards and eastwards in the Northern Hemisphere. The historical range of distribution includes most African countries and more recently, the Middle East; however, within the past decade, a dramatic expansion into the Balkans, eastern Europe, Russia, China and most of southern Asia has been experienced, with Indonesia and Singapore being some of the latest countries to experience outbreaks [11,12,13,14]. Epidemiological explanations for the highly rapid spread are mostly lacking. While a comprehensive genomic characterization of newly circulating strains is not yet available, the current knowledge on LSDV genetics demonstrates that genome rearrangements between field and vaccine strains are possible [15,16], with evidence to support this having occurred at least once in a contaminated vaccine batch, with subsequent use of this batch in the field leading to widespread dispersal [17]. In this context, understanding the unprecedented spread of LSDV is becoming increasingly complicated [18].

Solid evidence pertaining to the predominant transmission route of the virus is still scarce [19,20]. Indirect data extrapolated from epidemiological observations strongly suggest a correlation between summer months and outbreak frequency, which is indicative of arthropod involvement [21]. Seasonal patterns of spread have been found in Africa and countries in the Northern Hemisphere, pointing to the fact that different vectors and/or vector-assisted mechanisms can operate in different environments [12,22,23]. Experimental work in a laboratory setting demonstrated that African hard ticks and stable flies can mechanically transmit the virus from infected animals to naïve ones [24,25,26]. Other identified candidate vectors include mosquitoes, midges and Tabanidae fly species [27,28]. Contact transmission is considered to be ineffective for classical field isolates, whereas a recombinant vaccine-derived strain isolated from an active outbreak in 2017 (Saratov/2017) can spread without the involvement of arthropods [29]. Interestingly, the detection of LSD outbreaks in Russia caused by vaccine-derived recombinant isolates in cold months defies explanation via the vector-borne transmission theory only, requiring further investigation [18,30].

In this study, we extend our previous findings on the non-vector-borne transmission of Saratov/2017 to demonstrate infection of cattle fed on feed inoculated with this recombinant strain versus non-transmission when using a classical field strain (Dagestan/2015).

## 2. Results

### 2.1. Classical Strain, Dagestan/2015

The experimental animals in this group showed no fever, or other clinical signs, and PCR and ELISA results were all negative.

### 2.2. Recombinant Vaccine-like Strain, Saratov/2017

#### 2.2.1. Clinical Signs

**Figure 1:**
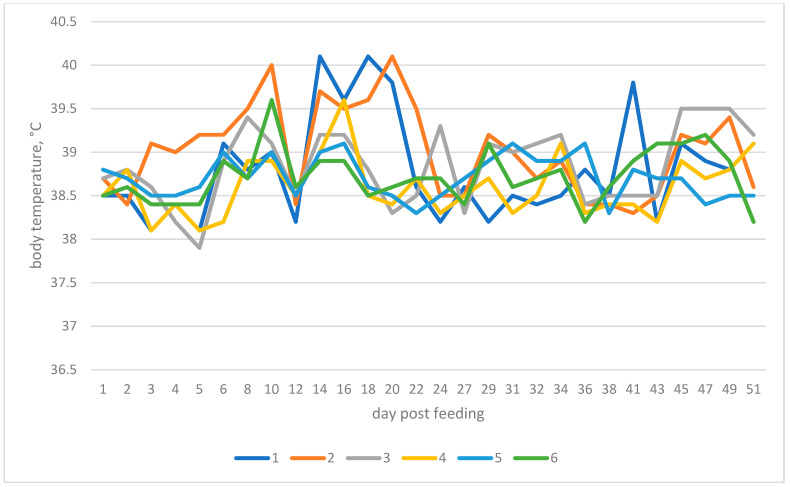
Changes in body temperature of animals exposed to inoculated bread feeding over time for the Saratov/2017 group (the numbers denote the assigned bull numbers).

**Bull №1.** Fever was erratic up to 40 °C (Figure 1). Edema and nasal discharge were observed at day 3.

**Figure 2:**
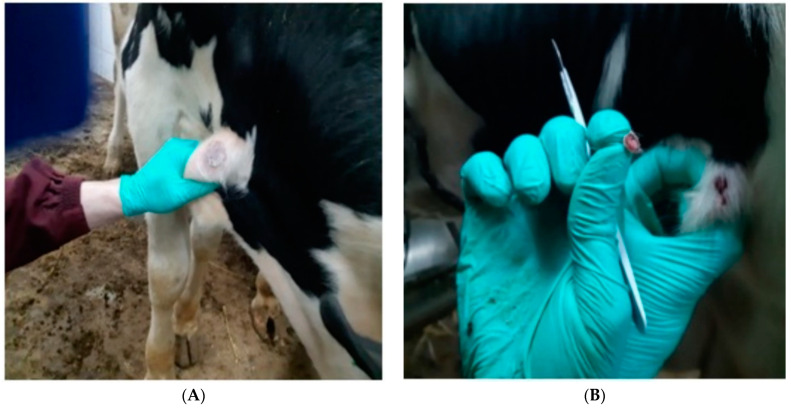
(**A**) Necrotised skin lesion (Bull №2) (**B**) Sequestrated skin lesion (Bull №2).

**Bull №2** (in-contact, negative control). An increased body temperature of up to 40 °C was observed at day 10 and day 20 (Figure 1). At day 13, a single lump measuring 0.5 × 0.5 cm was identified in the neck area. At day 15, a number of small lumps appeared on both sides of the neck. At day 19, a full-blown nodule (chicken egg-sized) developed on the animal’s right lateral side (Figure 2A). Nodules underwent further sequestration (Figure 2B). The sequencing of the virus from nodules confirmed the presence of Saratov/2017-specific sequences.

**Bulls №3, 5 and 6.** No clinical signs, i.e., no fever, nodules, edema, or nasal discharge were observed throughout the experiment.

**Bull №4.** At day 2, this animal started excreting a white nasal discharge lasting for 32 days. At day 44, a 0.5 × 0.5 cm lump appeared on the chin. At day 47, small lumps appeared on the neck. No fever was detected.

#### 2.2.2. Post-Mortem Examination

**Figure 3:**
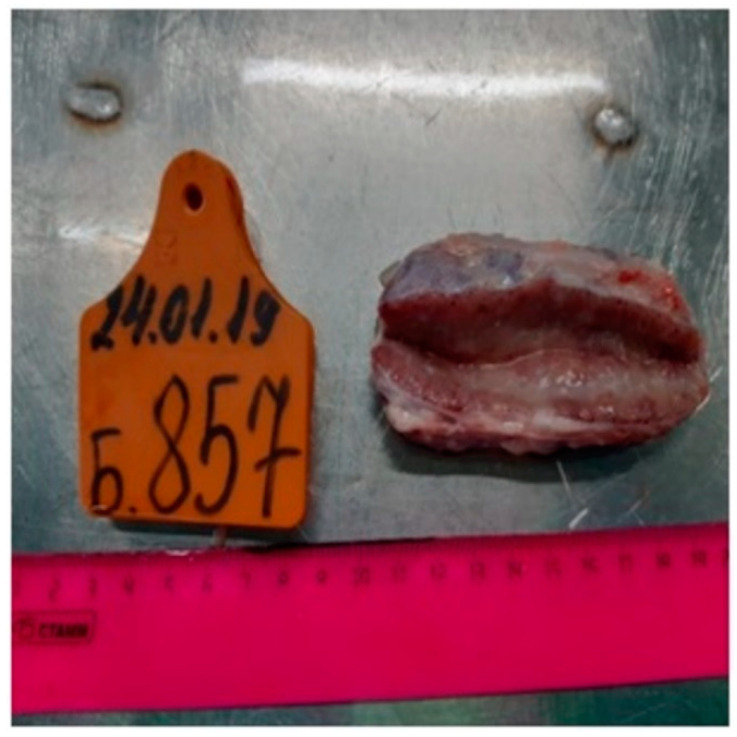
Prescapular node: edema and hemorrhages (Bull №1).

**Bull №1**—Post-pharyngeal and prescapular lymph nodes were enlarged with focal hemorrhages (Figure 3). The trachea and lungs were hyperaemic with multiple hemorrhages.

**Figure 4:**
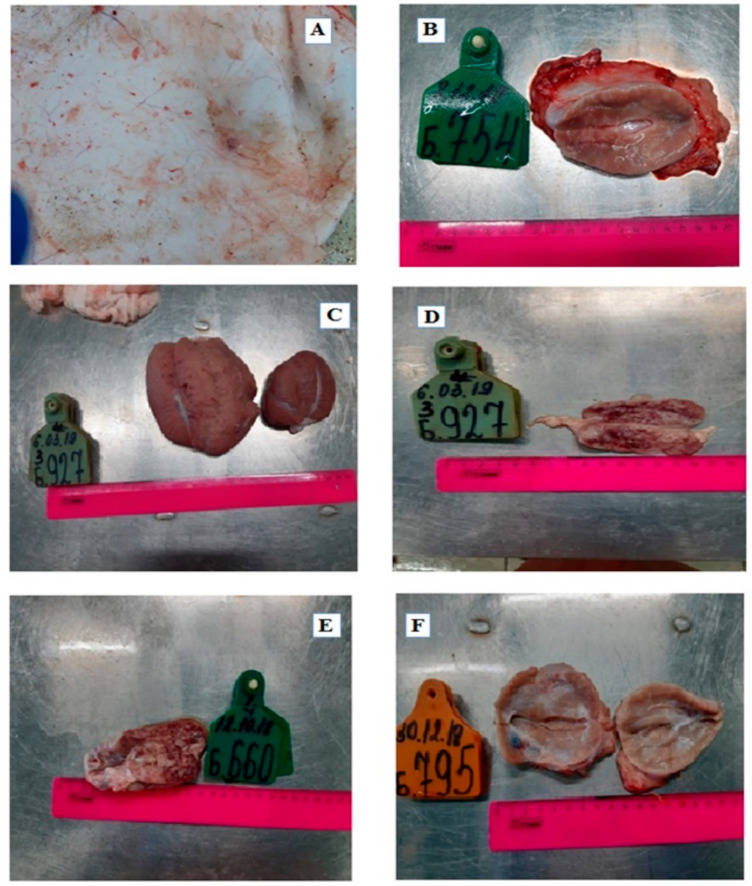
Pathologies observed in various organs. (**A**) The hypodermis of the skin (the arrows indicate a lesion) of Bull №2; (**B**) Prescapular node displaying hemorrhaging, Bull №2; (**C**) Testes: hyperaemic, Bull №3; (**D**) Mesenteric lymph node, Bull №3; (**E**) Mesenteric lymph node, enlarged, Bull №4; (**F**) Posterior pharyngeal lymph node enlargement of Bull №5.

**Bull №2**—There were lesions on the hypodermis of the skin of the groin and neck areas (Figure 4A), lymph nodes were enlarged with hemorrhages (Figure 4B), and the lungs were hyperaemic with a nodule in the upper lobe of one of them.

**Bull №3**—The testes were swollen and hyperaemic (Figure 4C), lymph nodes were enlarged with hemorrhages (Figure 4D), and the trachea and lungs were hyperaemic with multiple hemorrhages.

**Bull №4**—Lymph nodes were enlarged (Figure 4E).

**Bull №5**—Only the posterior pharyngeal lymph node was enlarged (Figure 4F).

**Bull №6**—Only the prescapular lymph node was enlarged and hyperaemic.

#### 2.2.3. PCR Testing

The results of PCR testing of blood samples and nasal swabs are shown in Table 1 and Table 2, respectively.

Viral genomic DNA was intermittently identified in Bulls №1–3, consistent with clinical signs. In Bull №1, LSDV DNA was once detected at day 16 of the trial. In nasal swabs, LSDV DNA was identified at day 24.

In Bull №2 (the in-contact, negative control animal), PCR-detection of viral DNA in its blood lasted 12 days, from days 18 to 29, whereas LSDV DNA was detectable in its nasal swabs for 23 days, from day 20 to 42.

In Bull №3, detectable viremia via PCR lasted 3 days from day 22 to 24. No detectable DNA was identified in nasal swabs.

In Bull №4, DNA was detected at days 41 and 45. Nasal swabs were positive at days 24, 29 and 45.

#### 2.2.4. ELISA Testing

**Scheme 1:**
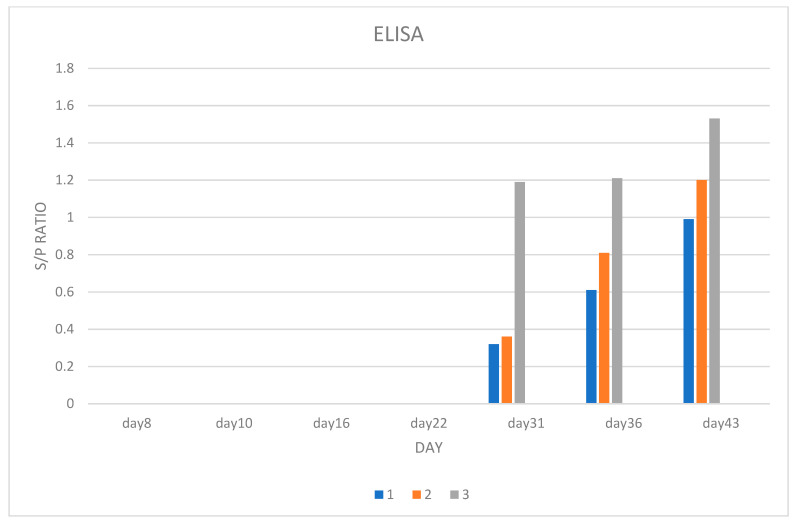
Bulls №1 and 3 were fed virus-inoculated bread, whereas Bull №2 was mock-fed.

ELISA results are provided in Scheme 1.

Only Bulls №1–3 displayed detectable seroconversion. The first detectable antibodies were observed at day 31 and increased until day 43. Bull №2, which did not receive virus via inoculated feed, displayed increasing levels of antibodies from day 31 onwards.

#### 2.2.5. Histological Examination

**Figure 5:**
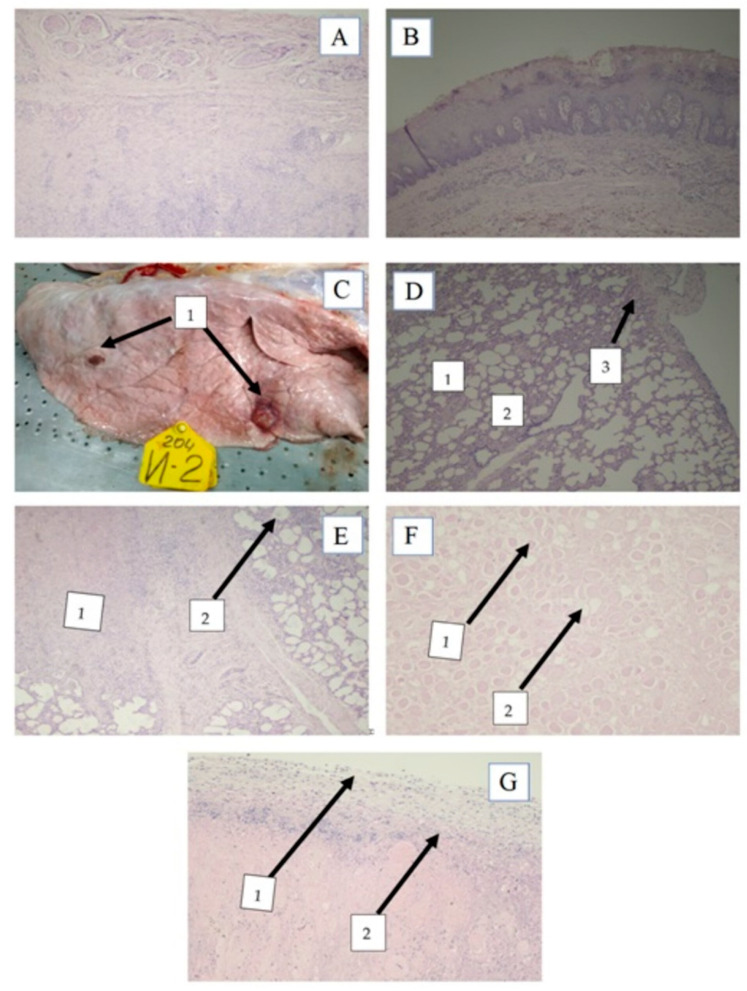
Histopathologic findings of lumpy skin disease virus in cattle tissue. (**A**) Subacute liquefactive (pus) necrosis in the derma. H and E staining, magnification X40; (**B**) Esophageal Necrotic Lesion. H and E staining, magnification X40; (**C**) Lobular pneumonia with multiple necrotic sites: 1—foci of necrosis; (**D**) Affected lungs. 1—Atelectases, 2—dislectases, 3—pleurite. H and E staining, magnification X40; (**E**) Focal purulonecrotic pneumonia; 1—necrosis, 2—thickened interalveolar septa; (**F**) Muscle dystrophy. 1—edema, 2—muscle fiber dystrophy. H and E staining, magnification X40; (**G**) The serous membrane. Focal serositis. 1—inflammation, 2—lymphocytic infiltrations. H and E staining, magnification X4.

Samples for histological analysis were derived from Bull №2 as confirmation of contact transmission. At the histological level, the skin lesion was surrounded by dense irregular connective tissue that is part of the skin derma. Under the derma are large infiltrations of lymphocytes and histiocytes, with an increasing concentration from the periphery to the center. Many cells of the lesion have undergone karyopiknosis and karyolysis (Figure 5A).

Examination of the gastrointestinal epithelium showed focal necrotic sites accompanied by damage to subepithelial tissue (Figure 5B).

Lungs demonstrated lobular pneumonia with necrotic sites, whereas at the histological level atelectasis and dislectases were observed (Figure 5C). The pulmonary pleurae and interalveolar septa were expanded and infiltrated with lymphocytes. Serous exudate within the lumen of bronchial tubes was present, with tube walls showing sclerotization (Figure 5D).

During the subacute inflammatory stage, we observed the expansion of interalveolar septa, peribronchovascular lymphocytic-histicytic infiltration and macrofocal purulonecrotic pneumonia. The pleura was expanded and infiltrated with lymphocytes. Most cellular nuclei already attained lytic or rhexis states (Figure 5E).

Histological examination of muscle tissue revealed thickening and lymphocytic infiltration in the intermuscular layers of connective tissues, edema and diffuse dystrophy of muscle fiber sheaves (Figure 5F). Serous lesions were observed in the abdominal cavity with lymphocytic infiltrations and hemorrhages (Figure 5G).

## 3. Discussion

The issue of LSDV transmission has been puzzling in many respects since no conclusive proof has yet been obtained as to the dominant mechanism, applicable to real field conditions both in tropical and northern climates [19,23]. Early experiments on LSDV transmission concluded that generalized infection is precipitated by only intravenous inoculation consistent with the emulation of an insect bite, while contact transmission is deemed ineffective and does not account for the fast-paced spread in a northerly, and subsequent easterly, direction of the virus in recent years [30,31]. Various hard tick species have also been implicated, as supported experimentally; however, these most likely only account for short-distance spread [25]. Experimental work involving laboratory-reared arthropods and mathematical modeling has demonstrated that the stable fly (*Stomoxys calcitrans*) and Culicoides midges are the most likely blood-sucking vectors for natural virus transmission, but testing of field-caught specimens, including species such as horse flies, during outbreaks has yet to provide definitive confirmation [25,28,32].

While attempts to achieve LSDV infection by classical field strains without using blood-sucking arthropods have been unsuccessful [33], field-isolated recombinant LSDVs with altered genotypic and phenotypic characteristics exhibit novel features enabling transmission from animal-to-animal without a dependency on insects [29,30]. Since the nature of contact transmission was not clear from our previous work [29], whether it was via the respiratory or the alimentary route, in this study we set out to provide the answer by conducting an experiment involving ingestion of feed in the form of brown bread soaked in virus-culture suspension. To contribute to a better understanding of the biological differences of currently circulating LSDV variants, a classical field isolate, Dagestan/2015, and a recombinant vaccine-derived strain, Saratov/2017, were compared in parallel.

In the group fed with bread soaked in culture containing strain Dagestan/2015, the experimental animals remained healthy and were PCR- and serologically-negative up to day 50 of the trial. These results agree with previous investigations, showing that in-contact transmission of classical LSDV strains is ineffective. Dagestan/2015, along with SERBIA/Bujanovac/2016, the Greek strain Evros/GR/15, the Bulgarian strain 210LSD-249/BUL/16, and the Warmbaths LW strain from South Africa are included in the group of classical field isolates, comprising cluster 1.2 [16,34,35,36]. Since these were the only known genotypes circulating in the field worldwide until 2017 [16], this explains why they were widely used in experiments in the past [9,31].

By contrast, Saratov/2017 is the first recombinant strain isolated in the field, comprising a genome most likely derived from a Neethling-type virus from cluster 1.1 as the major parent and a Kenyan KSGP vaccine virus as the minor parent from cluster 1.2 [15,17,36]. Importantly, recombinant LSDV variants are now dominating in outbreaks in Russia and causing outbreaks in China and Vietnam [14,18,37], while in India and Bangladesh the minor parental strain, a KSGP-like LSDV, is on the rise [38,39].

Two of the five experimental animals that received virus-inoculated feed in the Saratov/2017 group exhibited very mild clinical signs, which may be overlooked by inexperienced vets, and low and erratic detectability of virus genomes in blood and nasal secretions (Table 1 and Table 2, and Figure 1). Three animals (Bulls №4–6) were clinically healthy but Bull №4 was PCR-positive in blood and swabs. Previous experiments show that not all experimentally-inoculated animals succumb to LSDV infection [9,10]. Our findings reiterate this evidence. Bull №4 seemed to clear the virus without developing symptoms, serving as a subclinically ill animal, which is well-known for LSD [29,40]. The reasons behind acute, subacute and inapparent types of LSD are still elusive and constitute major gaps in the knowledge-base of the disease.

By contrast, Bull №2 in this group, which served as an in-contact negative control animal, became infected and displayed an even more severe disease presentation as compared to either of its flanking animals, Bull №1 or Bull №3. Infection in the in-contact animal was also evident at the histological level (Figure 5). Of note, Bull №2 (in-contact) became positive on day 18 with a Ct of 33.56. This onset is only two days after Bull №1 (soaked bread fed). The positivity of Bull №2 in the blood and swabs was stronger and longer as compared to the flanking animals and more distant ones. Moreover, Bull №2 was the first to have positive nasal swabs even before the animals of the soaked bread group. This is a very interesting observation that is challenging to explain logically in the light of the current knowledge on LSD virus transmission [19]. Since our experiment was carried out in the absence of arthropods, we will leave aside the vector-borne transmission route as a possible explanation and embark on alternative theories.

At day 18 and 20, all animals but Bull №2 tested negative in nasal swabs from each, which are normally a good source of virus. Since it takes time for virus titres to increase in the host, it is likely that at those time points virus loads in animals fed virus-soaked bread were lower than the assay detection limit, which caused the discrepancy. 

Importantly, the very rapid rise in body temperature may suggest Bull №2 was able to feed on some of the virus-inoculated bread left over from adjacent animals. However, this was not likely as the troughs were separated, and the animals were tethered to prevent cross-trough feeding. Another possible scenario is that some virus-soaked bread fell onto the floor and was then inadvertently kicked or pushed within reach of Bull №2. In any future trials of this nature, the investigators will endeavor to ensure that any possibility of in-contact negative control animals gaining direct access to virus-inoculated feed is excluded.

It is also possible that the virus was transmitted from the virus-fed animals to Bull №2 via the airborne route. Early work on LSD raised concerns that the virus can transmit without involvement of arthropods, but at a low level of efficiency [20] This is supported by outbreaks in northern latitudes occurring outside the optimal vector-weather conditions period or even during the freezing winter months [41]. This is further supported by the fact that the other capripoxviruses, sheeppox and goat poxviruses, and poxviruses in general, are contagious [42], and LSDV is no exception. In our experiment, some virus may have become aerosolized during the feeding process and the virus-containing aerosol droplets were then responsible for infecting Bull №2 (inoculated bread contained high virus titers). This possibility would be further supported if Bull №2 was genetically predisposed to infection or was immunocompromised, which cannot be excluded within the scope of this study. The problem associated with this theory is the lack of research correlating genetics with virus phenotype in vivo.

As more LSDV strains emerge, including recombinants with diverse patterns of recombination and parental strain contributions, additional studies will be needed to provide clarification on all possible aspects of LSDV transmission.

Despite the important knowledge gained from our study, we used only six animals per group, which may be deemed as a limitation. However, experiments published by Wolff et al. (2020 and 2022) [43,44], Babiuk et al. (2008) [9] and Tuppurainen et al. (2005) [45] utilised six to eight animals per group, whereas Carn and Kitching (1995) [31] used a few groups ranging from two to twenty-five animals each, and Aerts et al. (2021) [40] used two groups of five each. Our study with the current sample size generally falls in line with the cited research. Considering the fact that LSDV is able to induce subclinical infection [10,40], more animals per group are the better option, but this is cost-prohibitive and thus not practical. For this reason, more future studies with novel recombinant LSDV strains are needed to discover the extent to which various genetic lineages of LSDV can employ alternative transmission pathways.

Interestingly, in-contact transmission was detected in a previous study, but the results reported here cannot be used to confirm the results of the previous study, as a different route of infection was investigated here [29]. In this study, we obtained further confirmation that recombinant LSDVs, in particular, Saratov/2017, can employ alternative mechanisms for spread, whereas the transmission of classical field isolates, such as Dagestan/2015, depends largely on arthropod vectors, as transmission of these strains does not appear to occur in a vector proof setting. Our findings indicate that a non-vector-borne route of infection in cattle is more feasible for recombinant LSDVs than for classical field isolates [29].

A mild or subclinical disease presentation of animals infected with a recombinant virus in the field may enable low-level circulation of the virus between animals in a herd during a vector-free period, until favorable conditions set in for vector-assisted spread. This evidence provides a possible explanation for the LSD outbreaks in winter conditions in Russia in 2018–2019, caused by recombinant isolates. Since recombinant LSDVs are currently spreading in Eastern Asian countries, it is important to test other strains with different recombination patterns to verify the identified alternative modes of transmission. Clarifying the mechanisms by which classical and recombinant LSDVs overwinter in different climates will greatly improve control and eradication strategies.

Genomic rearrangements have profound effects on phenotypic characteristics of poxviruses [46,47]. Past studies hypothesized that all capripoxviruses arose by recombination from an ancestral strain [48]. This hypothesis acquired support in 2017 when recombinant LSDVs were discovered in the field [15]—however, how these current recombinants arose is still under debate, as there is evidence that they may have originated in culture during vaccine batch preparation [17]. Saratov/2017 is not the only recombinant strain detected in the field to date and strains arising from different recombinant events are still in circulation [18,49].

Overall, in this study we followed up on previous experiments where we had for the first time shown vector-free transmission of the recombinant strain, Saratov/2017, and here we proved that this strain, in contrast to the classical field strain, Dagestan/2015, can cause a mild disease via the alimentary route. Interestingly enough, the control animal did not receive virus-inoculated feed but contracted the virus by other means, which provides evidence that recombinant LSDV can be transmitted via alternative routes, as occurs for sheeppox and goatpox capripoxviruses. Further studies are currently underway to explore the degree to which other recombinant LSDV strains display contact infection. This information will help us better understand the relationship between phenotype and genotype of LSDV.

## 4. Materials and Methods

### 4.1. Viruses

The recombinant vaccine-derived strain, Saratov/2017 [15], and the classical field strain, Dagestan/2015, were used [35]. The strains cultured for this study were obtained from skin nodules on infected animals from previous experiments and were propagated as described [10].

### 4.2. Experimental Design

A total of six bulls were used in the experiment for each virus strain for assessing the potential for alimentary tract infection via ingestion of inoculated feed under biosafety level 3 and vector proof conditions. All animals in both experimental groups were 4–6 months old bulls of the Russian black pied breed, purchased from the same breeder. The bulls were tethered, but movement was not completely restricted and a degree of physical contact was possible between adjacent animals. The infection procedure was as follows: each bull was fed a piece of brown bread, weighing 100g and pre-inoculated with 5 cm^3^ of virus suspension of either strain containing 10^5^ TCD_50_/cm^3^ (day 1 of experiment). The virus for the soaking bread was harvested from a fresh cell culture exhibiting at least 50% cytopathic effects. The virus-soaked bread was given to animals within an hour of harvesting. No virus recovery from the soaked bread was carried out. The feeding was repeated with the virus-inoculated bread for five consecutive days (the first five days). If an animal did not finish eating its bread on any given day, the remainder was left in the individual troughs (not shared). The animals shared the same air space but were separated by metal railings and tethered so that one animal could not reach the other animal’s trough. The freedom of movement was such that the animal could eat only from its designated trough. One animal out of the six in either group (Bull №2) was a negative control, being fed the mock-inoculated bread, infiltrated with virus-free cell culture, but shared the same airspace with the others in its group. This negative control animal was located between Bull №1 and 3. Animals were screened each day once for clinical signs of LSD such as fever, lymph node enlargement or skin nodules. Nasal swabs and blood samples were collected every other day. Nasal swabbing was carried out at least 12 h post-feeding. Serum samples were collected at days 8, 16, 22, 31, 36, and 43 of the experiment. The total duration of the experiment was 50 days, after which animals were euthanised. The euthanasia protocol (permit number №7/12-05022019, provided as approval by the ethical committee of the FGBI ARRIAH) consisted of captive-bold penetration to desensitize the animals, followed by the injection of the muscle relaxant, Adilinum super, according to the manufacturer’s instructions (Federal Center for Toxicological, Radiation and Biological Safety, Kazan, Russia).

### 4.3. PCR and Sequencing

The assay targeting ORF44 of LSDV [50] and commercial Genesig^®^ Advance Kit (LSDV116 RNA polymerase subunit) following manufacturer’s recommendations were utilized for PCR testing. Total viral DNA was extracted using a QIAamp DNA Mini Kit (Qiagen, Hilden, Germany). All threshold values (Ct) below 38 were considered positive. Samples with low Cts (nodules) were subjected to sequencing at RPO30 and GPCR targets to confirm the identity of the strain to that used for infection as previously described [18].

### 4.4. ELISA

The ID Screen^®^ Capripox Double-Antigen Multi-species ELISA kit (ID Vet, Grabels, France) was used according to the manufacturer’s instructions for detecting antibodies to LSDV in sera [51]. The ELISA results were interpreted as follows: S/*N* ≥ 20%: positive.

### 4.5. Histology

Tissue sections for histological examination were removed in a volume of 1.0 cm^3^, followed by fixation in 10% neutral buffered formalin. The fixed specimens were processed using a TLP-720 tissue processor (Kreonika, Russia). Embedding was carried out via an ESD-2800 station (Kreonika, Russia). Sections were cut at a thickness of 0.005–0.008 cm^3^ using a RMD-3000 microtome station (Kreonika, Russia), followed by staining in hematoxilin and eosin. Light microscopy was performed using a Mikmed-6 microscope (Lomo, Russia).

## Figures and Tables

**Table 1:** PCR testing results of blood samples from animals fed virus-inoculated feed, showing Ct values—the Saratov/2017 strain group.

№ Animal	Day Post-Ingestion																
	15 *	16	18	20	22	24	26	28	29	31	34	36	38	41	42	44	45
№1	N	**33.76**	N	N	N	N	N	N	N	N	N	N	N	N	N	N	N
№2	N	N	**33.56**	**27.71**	**32.55**	**30.41**	**32.55**	**33.35**	**33.35**	N	N	N	N	N	N	N	N
№3	N	N	N	N	N	**33.51**	**34.99**	N	N	N	+	N	N	N	N	N	N
№4	N	N	N	N	N	N	N	N	N	N	N	N	N	**35.17**	N	N	**35.70**
№5	N	N	N	N	N	N	N	N	N	N	N	N	N	N	N	N	N
№6	N	N	N	N	N	N	N	N	N	N	N	N	N	N	N	N	N

* All samples before day 15 were negative for viremia when tested using real time PCR. N—negative by PCR. All Cts below 38 were considered positive. +: positive.

**Table 2:** PCR testing results of nasal swab samples from animals fed virus-inoculated feed, showing Ct values—the Saratov/2017 group.

№ Animal	Day Post-Ingestion																
	15 *	16	18	20	22	24	26	28	29	31	34	36	38	41	42	44	45
№1	N	N	N	N	N	**35.87**	N	N	N	N	N	N	N	N	N	N	N
№2	N	N	N	**36.17**	**25.80**	**26.20**	**29.26**	**30.44**	**31.72**	**32.34**	**37.19**	**28.85**	**34.05**	**35.19**	**32.82**	N	N
№3	N	N	N	N	**33.72**	**35.94**	N	N	N	N	N	N	N	N	N	N	N
№4	N	N	N	N	N	**34.16**	N	N	**33.35**	N	N	N	N	N	N	N	**35.65**
№5	N	N	N	N	N	N	N	N	N	N	N	N	N	N	N	N	N
№6	N	N	N	N	N	N	N	N	N	N	N	N	N	N	N	N	N

* All swab samples before day 15 were negative for virus when tested using real time PCR. N—negative by PCR. All Cts below 38 were considered positive.

## Data Availability

All data reported in this study are available within the manuscript.
